# Supplementary material for: WordCommentsAnalyzer: A windows software tool for qualitative research
Source: F1000Res. 2018 Sep 4;7:536. Originally published 2018 May 3. [Version 2] doi: 10.12688/f1000research.14819.2 (PMC6137411; doi:10.12688/f1000research.14819.2)
Supplement: Supplementary file 3 [file f1000research-7-17614-s0002.tgz › b2a79496-4ada-47c4-9682-a99ce867836d.zip!/Eight Iranian Health Journals/Journal of Knowledge and Health_23.docx]

© Journal of Knowledge and Health. This work is licensed under CC BY 4.0 (https://creativecommons.org/licenses/by/4.0/).

Volume & Issue: دوره 7، شماره 3: 1391

Title: رویکرد شناسه‌گزینی منطقی در تعیین مهم‌ترین اثرات متقابل ميان سطوح خونی اینترلوکین 6 و دو‌پلی‌مورفیسم تك‌نوكلئوتیدی ژن آن (IL-6-174 و IL-6-572) با برخی عوامل دیگر در ارتباط با پرفشاری خون

http://knh.shmu.ac.ir/index.php/site/article/view/70

مقدمه: هدف مطالعه حاضر، بررسی تأثيرات متقابل میان اینترلوکین 6 و نیز دوپلی‌مورفیسم‌های تك‌نوكلئوتیدی ژن آن ( IL-6-174 و IL-6-572) با برخی ديگر از عوامل شامل: جنس، سن، نمایه توده‌بدنی و سطح خونی قند، لیپید و پروتئين واكنشگر با ماده C در‌ارتباط‌با پرفشاری خون مي‌باشد. مواد و روش‌ها: در اين مطالعه مقطعي، داده‌های طرح "بررسی عوامل خطر نوین بیماری‌های غیرواگیر در جامعه شهری تهران" پژوهشکده علوم غدد درون‌ریز و متابولیسم دانشگاه علوم پزشکی شهید بهشتی، استفاده شد. برای شناسایی اثرات متقابل از رویکرد شناسه‌گزینی منطقی و برای تحليل از روش رگرسيون لجستيك استفاده شد و رابطه به شکل نسبت شانس تطبیق‌یافته بيان گردید. نتايج: مدل رگرسیونی برگزیده از تحليل داده‌های 126 مرد و 208 زن 18 ساله و بالاتر تهرانی، ضرایب معناداری را برای هریک از متغیرهای قندخون ≥ 126 mg/dl (01/0=P)، نمایه توده‌بدنی > 25 (03/0=P) و سن > 40 سال (001/0P<) و نیز برای ترکیب منطقی "کلسترول > 200 mg/dl+ سن > 40 سال + اینترلوکین 6> 29 pg/ml + قند خون < 126 mg/dl " (003/0=P) نشان داد. نتيجه‌گيري: این تحقیق با به‌کارگیری رویکرد شناسه‌گزینی منطقی نشان داد علاوه‌بر سن، نمایه توده‌بدنی و قند ‌خون ناشتای بالا، سطوح افزایش‌یافته اینترلوکین 6 در خون نیز در تعامل با کلسترول بالا، در شرایط سنی بالاتر و داشتن قند‌خون غير‌ديابتي، شانس پرفشاری خون را افزایش می‌دهند.

Title: بررسي الگوي آشکارسازي و وضعيت حمايت اجتماعي افراد HIV مثبت

http://knh.shmu.ac.ir/index.php/site/article/view/121

مقدمه: آشکارسازي وضعيت ابتلا به HIV از عوامل مهم در پيشگيري و کنترل اين بيماري به‌شمارمي‌آيد. هدف از پژوهش حاضر، تعيين الگوي آشکارسازي وضعيتHIV  و وضعيت حمايت اجتماعي درک شده افراد مبتلا می‌باشد. مواد و روش‌ها: 116 نفر از افراد HIV مثبت مراجعه‌کننده به مراکز مشاوره رفتاري به‌طور تصادفي انتخاب شدند. وضعيت حمايت اجتماعي با پرسش‌نامه خودايفاي نوربک و وضعيت آشکارسازي با دو سؤال بررسي گردید. نتايج: ميانگين سني افراد تحت مطالعه 7/7±7/36 سال بود و 4/72% از افراد نمونه مرد بودند. 2/42% از افراد، آلودگي از‌طريق رابطه جنسي، 44% ازطريق تزريق با سرنگ آلوده و 8/13% نيز ديگر موارد انتقال را ذکر نمودند. از ميان اين افراد، 1/62% وضعيتHIV  خود را آشکار کرده‌ بودند. در تحليل رگرسيون لجستيک، آشکارسازي، با متغيرهاي سن، جنس، تعداد CD4، تحصيلات بالاتر از ديپلم و حمايت اجتماعي کارکردي رابطه معناداری نشان داد. نتيجه‌گيري: بيش از نيمي از افراد، وضعيت خود را برای حداقل یکی از اعضای شبکه ارتباطی خود آشکار کرده بودند. آشکارسازي کمتر زنان مي‌تواند ناشي از نگرش جامعه درباره اين بيماري باشد. حمايت اجتماعي کارکردي مي‌تواند از عوامل مهم در آشکارسازي وضعيت ابتلا باشد. نتايج اين پژوهش لزوم توجه بيشتر به برنامه‌هاي آموزشي در افراد مبتلا و شبکه ارتباطي آن‌ها، درمورد اهميت آشکارسازي را نشان مي‌دهد.

Title: بررسی شیوع و علل گرایش به دخانیات در دانشجویان

http://knh.shmu.ac.ir/index.php/site/article/view/128

مقدمه: دانشجویان به‌عنوان افراد تحصیل‌کرده، الگوی مناسبی به‌عنوان فرد غیرسیگاری برای اطرافیان خود می‌باشند. پژوهش حاضر در سال 1389، به‌منظور بررسی علل شيوع و عوامل مؤثر بر مصرف دخانیات در بين دانشجويان دانشگاه‌هاي شاهرود انجام شده است. مواد و روش‌ها: این پژوهش يك مطالعه توصيفي- مقطعي است. داده‌ها ازطریق پرسش‌نامه‌اي حاوی 10 سؤال عمومی و 15 سؤال اختصاصی درزمینه مصرف دخانیات جمع‌آوري شده است. روايي پرسش‌نامه پس از تنظیم، مورد تأیید استادان و متخصصان فن قرار گرفته و 1800 نفر از دانشجویان دانشگاه‌های شاهرود به‌صورت خودایفا تکمیل كردند. نتایج: طبق نتایج به‌دست‌آمده میانگین سنی افراد 5/3±5/28 سال بود. 389 نفر (6/21%) از دانشجويان شركت‌كننده در اين مطالعه از دخانیات استفاده می‌کردند كه اكثريت آن‌ها 80% مذكر بودند. سیگار بیشترین ماده مصرفی بود، پس از آن به‌ترتیب 122 نفر (31%) از قلیان و 19 نفر (5%) از پیپ استفاده می‌کردند. از ميان عوامل مؤثر در گرايش به مصرف دخانيات؛ رابطه با دوستان سيگاري، استرس، دوري از خانواده و سرگرمی مهم‌ترين علل بودند. بین مصرف دخانیات با جنس، مقطع تحصیلی، وضعیت تأهل، محل سکونت، سطح تحصیلات والدین و ميزان درآمد آنان رابطه معناداری مشاهده گردید (001/0P<). نتيجه‌گيري: باتوجه به شیوع نسبتاً بالای مصرف دخانیات در بين دانشجویان، راه‌اندازی دفتر مشاوره و استفاده از همسالان آموزش‌دیده می‌تواند نقش بسزایی در کاهش مصرف دخانیات ایفا نماید.

Title: بررسي ارتباط كيفيت خواب و سلامت روان در دانشجويان ساكن خوابگاه‌

http://knh.shmu.ac.ir/index.php/site/article/view/131

مقدمه: اين مطالعه با هدف بررسي ارتباط كيفيت خواب و سلامت روان دانشجويان ساكن در خوابگاه انجام شده است. مواد و روش‌ها: پژوهش حاضر يك مطالعه‌ي مقطعي است كه بر‌روي 277 نفر از دانشجويان ساكن خوابگاه‌هاي دانشگاه علوم پزشكي تهران انجام شده است. روش نمونه‌گيري در اين مطالعه، تصادفي طبقه‌بندي شده بود. ابزارهاي مورد استفاده شامل، پرسش‌نامه دموگرافيك، پرسش‌نامه سنجش كيفيت خواب پيتزبورگ و پرسش‌نامه 28 سؤالي سلامت عمومي بودند. از آزمون‌هاي كاي اسكوار، اسپيرمن و رگرسيون لجستيك به‌منظور تجزيه‌و‌تحليل داده‌ها استفاده گرديد. نتایج: در پژوهش حاضر، شيوع كيفيت خواب نامطلوب، 3/73% (5/78- 1/68:CI 95/0) و شیوع سطح وضعیت نامطلوب سلامت روان، 3/34% (9/39- 7/28:CI 95/0) بود. ارتباط معناداري بین كيفيت خواب و وضعيت سلامت روان دانشجويان بود (001/0P<). نتيجه‌گيري: نتايج اين مطالعه نشان داد كه بين سلامت روان و كيفيت خواب دانشجويان ساكن خوابگاه ارتباط مستقيم و معناداري وجود دارد، بنابراين توصيه مي‌گردد برنامه‌های مداخله‌اي با هدف ارتقاي كيفيت خواب دانشجويان ساكن در خوابگاه، بر موضوع پيشگيري يا كاهش مشكلات بهداشت روان آن‌ها و آموزش اصول بهداشت خواب متمركز گردند.

Title: بررسي تأثير زنجبيل بر شدت تهوع و استفراغ پس از عمل جراحي

http://knh.shmu.ac.ir/index.php/site/article/view/133

مقدمه: تهوع و استفراغ پس از اعمال جراحي يکي از شايع‌ترين عوارض جانبي همراه با جراحي است. هدف اين مطالعه بررسي تأثير زنجبيل بر شدت تهوع و استفراغ پس از عمل جراحي مي‌باشد. مواد و روش‌ها: در اين کارآزمايي باليني دوسوکور تعداد 160 بيمار واجد شرايط به‌صورت تصادفي و به‌روش بلوک چهارتايي در دو گروه مداخله و شاهد قرار داده شدند. گروه مداخله، 4 کپسول محتوي250 ميلي‌گرم زنجبيل و گروه شاهد 4 کپسول دارونما را يك ساعت قبل از عمل جراحي دريافت نمودند. شدت تهوع و دفعات استفراغ در 2، 4 و 6 ساعت پس از جراحي با استفاده از مقياس بصري (VAS) سنجش شد. مقايسه نمرات بين دو گروه با استفاده از آزمون تي مستقل و آزمون من ويتني يو انجام شد. نتايج: ميانگين نمره تهوع دو ساعت اول بعد از عمل در گروه مداخله به‌طور معناداري پايين‌تر بود (04/0P=). ميانگين نمرات تهوع در ساعات 4 و 6 بعد از عمل نيز در گروه مداخله پايين‌تر از گروه شاهد بود، اما اختلاف آماري معناداري در دو گروه در هيچ‌يک از اين ساعات وجود نداشت. ميانگين دفعات تهوع در گروه مداخله در ساعات دوم، چهارم و ششم بعد از عمل پايين‌تر از گروه شاهد بود، اما اين اختلاف فقط در ساعت دو بعد از عمل به‌صورت مرزي (05/0P=) معنادار بود. شدت استفراغ در هيچ‌يک از ساعات در دو گروه مداخله و شاهد اختلاف معناداري نداشت. نتيجه‌گيري: استفاده از زنجبيل در پيشگيري از تهوع پس از اعمال جراحي مؤثر است. زنجبيل مي‌تواند به‌عنوان يک داروي ضدّ‌تهوع ايمن بعد از اعمال جراحي استفاده شود.

Title: ارزيابي طرح پزشك خانواده براساس شاخص‌هاي سلامت خانواده

http://knh.shmu.ac.ir/index.php/site/article/view/141

مقدمه: تیم پزشک خانواده وظیفه ارائه تمام خدمات ازجمله بهداشت مادر و كودك را برعهده‌دارند. این مطالعه با هدف ارزیابی تأثير اجرای طرح پزشک خانواده بر شاخص‌هاي سلامت خانواده در مناطق شهری انجام شده است. مواد و روش‌ها: در این پژوهش، جمعیت 36000 نفری سه شهر استان آذربایجان شرقی انتخاب و میزان‌های پوشش تنظیم خانواده زوجین در سال‌های 81 تا 87 به استناد آمار و نیز در بعد کیفی، پرونده 600 زوج تحت پوشش به‌‌روش تصادفی، سهمیه‌ای و خوشه‌ای از بين پرونده‌های خانوار انتخاب و مستندات آن بررسي شد. نتايج: با اجرای طرح، تعداد پزشک و ماما در این مراکز دو برابر شده‌اند. میزان پوشش از 7/39% به 2/66% افزایش يافت و روش‌های پیشگیری کاندوم افزایش ده درصدی و قرص و آی‌یودی کاهش 6 درصدی را نشان می‌دهند. با وجود اين تعداد متولدين در مدت هفت سال تقريباً ثابت مانده است. معاینات ادواری زنان و نمونه‌برداری پاپ اسمیر افزایش قابل توجه و معناداری (05/0P<) را نشان مي‌دهد. نتیجه‌گیری: اجرای طرح پزشك خانواده منجر‌به موفقيت نسبي خدمات سلامتي براي خانوارها ازجمله معاينه ادواري زنان و نمونه‌برداري پاپ اسمير شده است، لذا بازنگری در شرح وظایف، نحوه ارزیابی عملكرد تيم پزشك خانواده توصیه می‌گردد.

Title: اثر بربرین هیدروکلراید بر آسیب بیضه‌ای در موش‌های صحرایی دیابتی‌شده با استرپتوزوتوسین

http://knh.shmu.ac.ir/index.php/site/article/view/145

مقدمه: دیابت ملیتوس با ایجاد استرس اکسیداتیو، آسیب‌های متابولیسمی متعددی در بیضه‌ها ایجاد می‌كند. این مطالعه با هدف پیشگیری از عوارض اکسیداتیو ناشی از دیابت با به‌کارگیری داروی بربرین هیدروکلراید که دارای خواص آنتی‌اکسیدان و جمع‌کننده رادیکال‌های آزاد می‌باشد، انجام گردید. مواد و روش‌ها: در این مطالعه 32 سر موش صحرایی نر از نژاد ویستار، با محدوده وزنی 220 تا 240 گرم، انتخاب و به 6 گروه تقسیم شدند: 1- گروه شاهد، 2- تیمارشده با بربرین mg/kg100، 3- گروه شاهد تیمارشده با بربرین mg/kg 50، 4- گروه دیابتی، 5- گروه دیابتی تیمارشده با بربرین mg/kg 100 و 6- گروه دیابتی تیمارشده با بربرین mg/kg 50. دیابت ازطريق تزریق درون‌صفاقی استرپتوزوتوسین (mg/kg 50) ایجاد گردید. تیمار با بربرین با دوز mg/kg50 و 100 به مدت 6 هفته به‌صورت گاواژ انجام شد. در ابتدای هفته پنجم، از نمونه‌ها خون‌گیری به‌عمل آمده و فاکتورهای بیوشیمیایی خون و مقاطع بیضه‌ها مورد ارزیابی هیستولوژیک قرار گرفت. نتایج: نتایج مطالعه نشان می‌دهد كه دیابت با ایجاد استرس‌های اکسیداتیو و تولید رادیکال‌های آزاد، باعث کاهش سلول‌های اسپرماتوگونی و سرتولی، کاهش روند اسپرماتوژنز و نیز کاهش تعداد اسپرم و فعالیت اسپرم گردیده است. در نمونه‌های تیمارشده با بربرین این عوارض بهبود یافته، همچنین افزایش در تعداد اسپرم، تحرک اسپرم، سلول‌های رده اسپرم‌ساز و تعداد i سلول سرتولی نیز مشاهده گردید. به‌علاوه در گروه‌های درمانی قطر لوله اسپرم‌ساز افزایش یافته و ضخامت غشاي پایه کاهش یافت. نتیجه‌گیری: بربرین با توانایی کاهش قندخون، پیشگیری و بهبود استرس‌های اکسیداتیو، در بهبود آسیب‌های بیضه‌ای ناشی از دیابت نقشی درمانی ایفا می‌کند.

Title: اثر تجویز دراز‌مدت ملاتونین در درمان آسیب‌های بیضه‌ای ناشی

http://knh.shmu.ac.ir/index.php/site/article/view/149

مقدمه : بیماری دیابت به‌سبب ایجاد استرس اکسیداتیو، نقش مؤثري در پیدایش آسیب‌های متابولیسمی دارد. هدف مطالعه حاضر، بررسي اثر ملاتونین باتوجه به خواص آنتی‌اکسیدان آن در درمان آسيب‌های بیضه‌ای ناشی از دیابت مي‌باشد. مواد و روش‌ها: در این مطالعه 24 سر موش صحرایی نر از نژاد ویستار، با محدوده وزنی 200 تا 220 گرم، انتخاب و به سه گروه شاهد، دیابتی و درمانی تقسیم شدند. دیابت ازطریق تزریق درون‌صفاقیmg/kg 55 استرپتوزوتوسین (STZ) ایجاد شد و گروه درمانی (دیابتی + ملاتونین) پس از دو ماه دیابتی‌شدن، به‌مدت 5 هفته داروی ملاتونین با دوز mg/kg 10 را به‌روش گاواژ دریافت نمودند. در ابتدای هفته پنجم از نمونه‌ها خون‌گیری به‌عمل‌آمد و فاکتور‌های بیوشیمیایی و هورمونی خون و همچنین مقاطع بیضه‌ها مورد ارزیابی هسیتولوژیک قرار گرفت. نتایج: دیابت با ایجاد استرس‌های اکسیداتیو و تولید رادیکال‌های آزاد باعث کاهش سلول‌های اسپرماتوگونی و سرتولی، کاهش روند اسپرماتوژنز تعداد اسپرم و فعالیت اسپرم گردیده، میزان هورمون‌های انسولین و تستوسترون را نیز کاهش می‌دهد. تجويز ملاتونین، تغییرات مورفوروژیک ایجادشده را به‌طور معناداری کاهش و میزان هورمون انسولین و تستوسترون پلاسما را افزایش می‌دهد. نتیجه‌گیری: ملاتونین با توانایی پیشگيری و بهبود استرس‌های اکسیداتیو، در بهبود آسیب‌های بیضه‌ای ناشی از دیابت نقشی درمانی ایفا می‌کند.
